# Biodegradable Periodic Mesoporous Organosilica (BPMO) Loaded with Daunorubicin: A Promising Nanoparticle‐Based Anticancer Drug

**DOI:** 10.1002/cmdc.201900595

**Published:** 2020-02-11

**Authors:** Ngoc Xuan Dat Mai, Albane Birault, Kotaro Matsumoto, Hanh Kieu Thi Ta, Soontaree Grace Intasa‐ard, Kendall Morrison, Phan Bach Thang, Tan Le Hoang Doan, Fuyuhiko Tamanoi

**Affiliations:** ^1^ Center for Innovative Materials and Architectures (INOMAR) Vietnam National University-Ho Chi Minh City Ho Chi Minh City 721337 Vietnam; ^2^ Faculty of Physics and Engineering Physics, University of Science Vietnam National University Ho Chi Minh City 700000 Vietnam; ^3^ Faculty of Materials Science and Technology, University of Science Vietnam National University Ho Chi Minh City 700000 Vietnam; ^4^ Institute for Integrated Cell-Material Sciences (ICeMS) Institute for Advanced Study Kyoto University Kyoto 606 8501 Japan; ^5^ TAE Life Sciences Drug Development Division Santa Monica, CA 90404 USA

**Keywords:** Biodegradable periodic mesoporous organosilica, Daunorubicin, Tetrasulfide-based nanoparticles, Tumor spheroid and chicken egg tumor models

## Abstract

Biodegradable periodic mesoporous organosilica (BPMO) nanoparticles have emerged as a promising type of nanocarrier for drug delivery, given the biodegradable feature is advantageous for clinical translation. In this paper, we report synthesis and characterization of daunorubicin (DNR) loaded BPMO. DNR was loaded onto rhodamine B‐labeled BPMO that contain tetrasulfide bonds. Tumor spheroids and chicken egg tumor models were used to characterize the activity in biological settings. In the first experiment we examined the uptake of BPMO into tumor spheroids prepared from ovarian cancer cells. BPMO were efficiently taken up into tumor spheroids and inhibited their growth. In the chicken egg tumor model, intravenous injection of DNR‐loaded BPMO led to the elimination of ovarian tumor. Lack of adverse effect on organs such as lung appears to be due to excellent tumor accumulation of BPMO. Thus, DNR‐loaded BPMO represents a promising nanodrug compared with free DNR currently used in cancer therapy. OK

## Graphical abstract

**Rhodamine B‐labelled** biodegradable periodic mesoporous organosilica (BPMO) containing tetrasulfide units were investigated by tumor spheroid and chicken egg models. These models are affordable, time‐saving and easy to scale up approaches for the evaluation of drug delivery systems such as BPMO nanoparticles. The results show excellent tumor accumulation of BMPO and dramatic tumor inhibition in the presence of daunorubicin‐loaded BPMO.

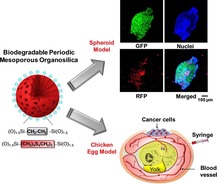

## Introduction

Advance in Nanotechnology led to the development of a variety of nanoparticle based anticancer drugs. Biodegradable periodic mesoporous organosilica (BPMO) nanoparticles represent a new class of mesoporous silica nanomaterials (MSN) that have attracted considerable attention as a promising vehicle in drug delivery.^1^, ^2^, ^3^, ^4^, ^5^ While MSN are synthesized by classical sol‐gel reaction of tetraethyl orthosilicate (TEOS), BPMO use bridged organosilane precursors that enable homogeneous and covalent incorporation of organic moieties. In particular, this approach results in the uniform distribution of biodegradable bonds into the silylated framework. These bonds include disulfide and tetrasulfide bonds that are sensitive to reducing conditions.^6^, ^7^, ^8^, ^9^ In addition, BPMO with protease sensitive bonds have been synthesized.^10^ BPMO possess all the advantageous features of pure inorganic MSN such as high surface area, large pore volume and fine control of the pore diameter.^11^, ^12^ On the other hands, the organic groups confer degradability under biorelevant conditions thus contributing to the issue of safety of nanomaterials during clinical use. Uptake of BPMO into cancer cells and delivery of anticancer drugs such as camptothecin and doxorubicin have been reported.^13^, ^14^, ^15^

To further evaluate the potential of BPMO to deliver anticancer drugs, we synthesized daunorubicin (DNR)‐loaded BPMO. DNR is an anthracycline antibiotics widely used for the treatment of a variety of cancers.^16^, ^17^, ^18^ Recent excitement in the AML therapy concerns liposomal formulation of daunorubicin and cytarabine (CPX‐351).^19^ Various liposomal formulation of DNR have been developed against a number of cancer including non‐small cell lung cancer and glioma.^20^, ^21^

We synthesized and characterized the activity of DNR‐loaded BPMO by employing two different tumor models. One of them uses tumor spheroids that are generated by culturing cancer cells in a three‐dimensional array.^22^ Another is chicken egg tumor model (chorioallantoic membrane (CAM) assay) that provides a simple and versatile system.^23^, ^24^, ^25^, ^26^ In this model, the tumor is formed 3–4 days after transplantation of human cancer cells owing to incomplete development of the immune system.^27^, ^28^ Meanwhile, the rich angiogenic system that is fully developed in the CAM membrane provides abundant nutrient.^29^ The newly formed tumor closely resembles one with human tumor pathology. Both systems are amenable for use with patient‐derived tumors; patient‐derived tumor can be used to produce tumor organoids and also to develop tumor inside the chicken eggs.

We first describe the synthesis and characterization of tetrasulfide‐based BPMO nanoparticles. Degradability of BPMO under reducing conditions in phosphate buffered saline (PBS) and in simulated body fluid (SBF) solutions was demonstrated. DNR,^18^, ^30^ a member of anthracycline family, is loaded onto BPMO after treatment with NaHCO_3_. DNR‐loaded BPMO inhibits tumor spheroid growth. In the chicken egg tumor model, DNR−BPMO eliminate the tumor after intravenous injection into the chicken egg tumor model. These results point to the promising features of DNR‐loaded BPMO as a chemotherapeutic drug.

## Results and Discussion

### Synthesis and characterization of BPMO nanoparticles

Fluorescent BPMO nanoparticles were synthesized by the sol‐gel method using two organoalkoxysilane precursors one of which includes bridged tetrasulfide units. The covalent incorporation of the tetrasulfide bonds makes the nanoparticles degradable under reducing conditions. Cetyltrimethylammonium bromide (CTAB) was used as a structure directing agent to promote pore network formation in the nanostructure. Nanoparticles were functionalized with rhodamine B isothiocyanate (RBITC) for tracking. They were also surface modified with phosphonate moiety (as described in the Experimental Section) to facilitate dispersion and prolonged blood circulation.

**Figure 1:**
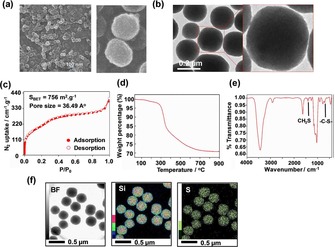
Characterization of BPMO: a) SEM images, b) TEM images, c) N_2_ adsorption‐desorption isotherm, d) Thermogravimetric analysis, e) FT‐IR, f) elemental mapping: Si and S.

Scanning electron micrographs (SEM) show homogeneous spherical nanoparticles with an average diameter of 200 nm in agreement with dynamic light scattering (DLS) results (Figure 1a and Figure S1, Supporting Information). The rough surface of particles is due to the presence of the pore network. Detailed structure of BPMO was analyzed by transmission electron microscopy (TEM). As shown in Figure 1b, the spherical shape of nanoparticles was confirmed and the pore network was clearly observed. To further demonstrate the featured pore structure, nitrogen adsorption‐desorption isotherm was performed (Figure 1c). BPMO shows the Type IV isotherm, indicating the presence of a mesoporous structure, which is consistent with TEM results. The Brunauer‐Emmett‐Teller (BET) surface area was 756 m^2^ g^−1^, the pore volume was 0.53 cm^3^ g^−1^. According to the Barrett‐Joyner‐Halenda (BJH) theory, the pore diameter was calculated to be approximately 36.5 Å.

To determine the presence of organic groups bridged into the hybrid framework, BPMO have been analyzed by thermogravimetric analysis (TGA) (5 °C/min up to 900 °C) on the nanoparticles after CTAB extraction (Figure 1d). A major weight decrease, observed around 350 °C, can be attributed to the decomposition of organic components. The thermal degradation behavior took place in a broadened temperature range up to 700 °C. Nearly 30 % of organic bridging groups were degraded, indicating significant condensation of the two silane precursors. To further investigate the chemical composition of BPMO, FT‐IR and elemental mapping analyses were carried out (Figure 1e and f). FT‐IR of the BPMO shows C−H bond at 2925, 1269, 909 cm^−1^ and C−S bond at 696 cm^−1^_,_ indicating the presence of thioether‐bridged in the framework^31^, ^32^ (Table S1, Supporting Information). The presence of C−S bond was also proven by Raman spectroscopy. It can be seen in Figure S2 that C−S bond was detected at 646 cm^−1^ while S−S bond was observed at 481 cm^−1^.^33^, ^34^, ^35^ Furthermore, elemental mapping of the BPMO provides clear evidence of the uniform distribution of sulfur atoms (Figure 1f) within the porous wall. The results suggest the successful embedding of organoalkoxysilane precursors within the BPMO network during the sol‐gel reactions.

**Figure 2:**
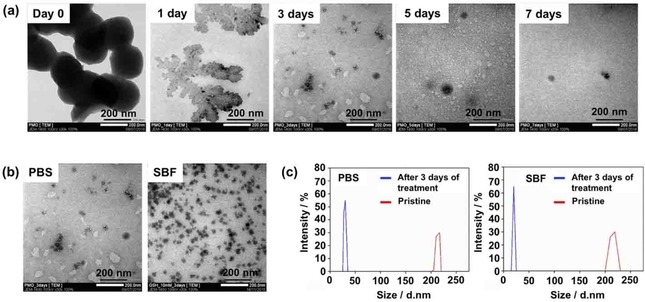
In vitro degradation of BPMO: a) TEM of degraded BPMO after incubating in PBS with GSH (10 mM) for various times. b) TEM of BPMO after incubating with GSH (10 mM) in PBS or SBF for 3 days. c) DLS measurements confirmed the average size of pristine BPMO and degraded fragments after 3 days of treatment.

Degradation of BPMO was investigated by treating with glutathione (GSH, 10 mM). As a first step to evaluate BPMO degradation, we dispersed BPMO in PBS as well as in SBF (simulated body fluid) solution containing GSH. The incubation was up to 7 days at room temperature and the degraded products were examined by TEM (Figure 2a). As can be seen, the spherical BPMO nanoparticles were decomposed after 3 days and most of the nanoparticles were converted into small spherical materials with a size range of 10–20 nm. By day 7, nanoparticles were almost completely degraded. Interestingly, a large structure was detected at day 1, which may represent an intermediate collapsed structure.

A similar degradation profile was observed when BPMO were incubated in SBF solution containing GSH (Figure 2b). Less destruction of BPMO was observed in SBF compared to that in PBS. This was presumably due to the influence of ions in the SBF solution which can interfere with the interaction between GSH and the tetrasulfide bond in the porous structure of BPMO. DLS measurements were consistent with the TEM micrographs as shown in Figure 2c.

For comparison, degradation of purely inorganic MSN nanoparticles (synthesized under similar conditions to those used for BPMO synthesis but using TEOS as a silane source) in PBS containing GSH (10 mM) was evaluated by TEM over 7 day‐period. The results shown in Figure S3 (Supporting Information) reveal that the nanoparticles remain intact during this period with respect to morphology and well‐ordered structure. Thus, MSN and BPMO differ in their degradability.

Daunorubicin (DNR) was loaded onto BPMO by first activating the BPMO with a 0.1 M NaHCO_3_ solution. Activated BPMO were suspended and DNR solution was added. The suspension was stirred overnight and DNR‐loaded BPMO were collected by centrifugation. The loading efficiency was analyzed by fluorescence measurements of the supernatants to determine the mass of encapsulated DNR. The calculated drug loading capacity was 12.04 weight %.

### Evaluation of BPMO using tumor spheroid model

**Figure 3:**
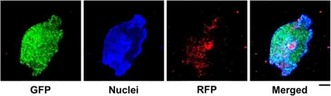
Uptake of rhodamine B‐labeled BPMO into 3D ovarian tumor spheroids after 18 h incubation. Scale bar: 100 μm.

To examine the behavior of free BPMO and DNR‐loaded BPMO in biological settings, we first used the tumor spheroid model. This model reproduces a three dimensional (3D) avascular multicellular tumor. It is a promising method for the evaluation of tumor penetration and cytotoxicity of drug nanoformulations.^22^, ^36^, ^37^ Green fluorescent protein (GFP) expressing human ovarian cancer cells (OVCAR8) were grown on a spheroid forming plate for 7 days to generate tumor spheroids. Spherical mass of cancer cells was produced as shown in Figure 3. The tumor spheroids were then incubated with rhodamine B‐labeled BPMO overnight. After washing, tumor spheroid nuclei were stained with Hoechst dye and the systems were observed by confocal microscopy. As shown in Figure 3, red fluorescence of the nanoparticles overlapped with GFP of tumor spheroids. These results demonstrate efficient uptake of BPMO into tumor spheroids.

**Figure 4:**
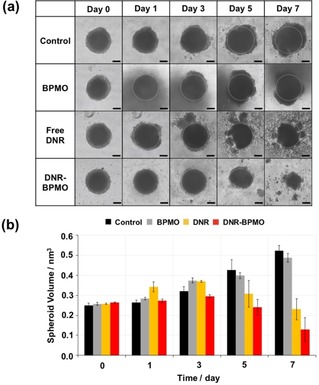
a) Optical image of ovarian tumor spheroid and (b) calculated spheroid volume after treatment with no injection (Control), free BPMO, free DNR or DNR−BPMO over a period of 7 days. Scale bar: 100 μm. Error bars show standard error.

Next, DNR was loaded into the BPMO (DNR−BPMO) and evaluated in the tumor spheroid model. OVCAR8‐based tumor spheroids were incubated with BPMO, free DNR (1.2 μg) or DNR−BPMO (equivalent concentration of 1.2 μg DNR) and the spheroid tumor growth was assessed for 7 days. As shown in the optical images in Figure 4a, spheroid growth inhibition was observed in the 3D tumors incubated with free DNR solution or with DNR−BPMO. Meanwhile, the tumor size of the control (no injection) and BPMO groups gradually increased, confirming that BPMO nanoparticles do not have inherent cytotoxicity on OVCAR8 tumor spheroids. Conversely, after one week, treatment with DNR−BPMO resulted in a tumor reduction of 51.1 % of its initial volume (Figure 4b). Incubation with free DNR also led to significant tumor growth repression (Figure 4b). The tumor volume shrank by 10.6 % of its initial volume after 7 days. Interestingly, the tumor volume in the free DNR and DNR−BPMO treated groups was 44.1 % and 24.7 %, respectively, compared to the control sample on day 7. It is noteworthy that the same tendency was observed when lower concentrations of drug were tested (Figure S4 Supporting Information).

### Evaluation of BPMO using chicken egg tumor model

**Figure 5:**
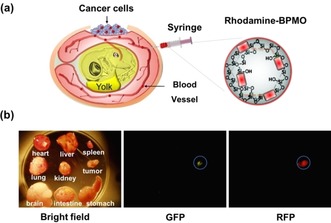
a) Chicken egg tumor model. OVCAR8 tumor produced by transplanting ovarian cancer cell expressing GFP. Rhodamine B‐labeled BPMO (0.2 mg.100 μL^−1^) were intravenously injected into chicken egg blood vessel. b) Bright field and fluorescence images indicate the preferential accumulation in the tumor of BPMO.

We then employed the chicken egg tumor model (the CAM assay) for further characterization of BPMO. This was used to evaluate tumor targeting capability of BPMO. BPMO were injected intravenously into fertilized chicken eggs with ovarian tumors produced by transplanting OVCAR8 cells (Figure 5a).

**Figure 6:**
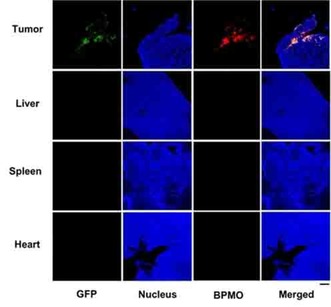
Confocal images of the accumulation of BPMO in the chicken egg embryo. Red fluorescence of BPMO was detected in tumor while no detection on other organs: liver, spleen and heart. Tumor and organs were collected 2 days after injection. Scale bar: 100 μ m.

Following 3 days after injection the tumor as well as various organs from chick embryo were dissected out and observed by fluorescent stereomicroscopy. As shown in Figure 5b, red fluorescence, from the rhodamine B‐labeled BPMO, was detected in tumor and shown to overlap with the green fluorescence of GFP cancer cells. Red fluorescence was not detected in any of the organs including liver, spleen, heart, kidney, intestine, brain, lung and stomach. This preferential tumor accumulation was further confirmed by mounting sections of the tumor and the different organs on a slide and examining them using confocal microscopy (Figure 6). As expected, red fluorescence signal once again overlapped with the GFP of the cancer cells. In contrast, red fluorescence was not detected in other organs (Figure 6).

**Figure 7:**
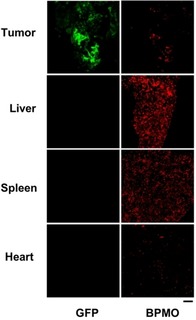
Confocal images of the accumulation of positively charged BPMO surface modified with amine groups (N‐(2‐aminoethyl)‐3‐aminopropyltrimethoxysilane) in the chicken egg embryo. Tumor and organs were collected 2 days after injection. Scale bar is 100 μ m.

The preferential accumulation of BPMO in the tumor appears to be due to negative surface charge of BPMO (zeta potential −31.27 mV), as we carry out phosphonate modification. We synthesized positively charged BPMO (zeta potential 22.53 mV) and this was intravenously injected into the chicken egg. As shown in Figure 7, we observed accumulation also in liver and spleen. Thus, surface charge is critical for biodistribution of BPMO.

**Figure 8:**
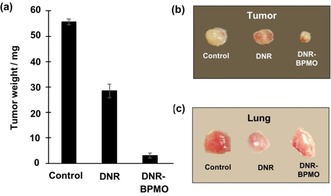
a) Tumor elimination of DNR−BPMO and free DNR. Comparison of (b) tumor and (c) lung from chicken egg embryos with no injection (Control) or 3 days after injection of free DNR and DNR−BPMO.

Finally, DNR‐loaded BPMO (DNR−BPMO) were intravenously injected into the chicken eggs to evaluate the effects on tumor growth. 3 days after injection of the nanoparticles the weight of the tumor was measured and the tumor photographed (Figure 8a,8b and Table S2). When DNR−BPMO were injected, the tumor size was found to be 5.4 % of the tumor of the no injection control, highlighting around 95 % of tumor eradication by using the drug‐loaded nanoparticles. Furthermore, tumor growth inhibition was significantly higher than that observed with free DNR. Figure 8c shows lung after injection of DNR‐loaded BPMO or free DNR. The results show the appearance of lungs after DNR−BPMO injection differs from those after free DNR injection and is more closely aligned with the control.

In this paper, we have reported synthesis and characterization of biodegradable periodic mesoporous organosilica (BPMO). To increase dispersion in solution, we surface modified with phosphonate derivatives. The BPMO nanoparticles contain tetrasulfide bonds, which can be degraded in a reducing environment. In fact, we showed that BPMO are degraded completely after incubation with glutathione in PBS or in SBF. Further experiments are needed to investigate BPMO degradation in biological systems. Characterization of BPMO included N_2_ adsorption‐desorption, thermogravimetric analysis, FT‐IR and electron microscopies. In particular, we carried out elemental mapping to show the presence of sulfur. Raman spectroscopy demonstrated covalent incorporation of sulfur into the framework.

The anthracycline daunorubicin could be efficiently loaded onto BPMO. By using tumor spheroids as well as chicken egg tumor model, we showed that DNR‐loaded BPMO exhibit tumor growth inhibition. Comparison with free DNR in the chicken egg model revealed that the nanoformulated DNR has less adverse effect, as seen by the lack of adverse effect on organs such as lung. A similar observation was made with liver and heart. Thus, BPMO formulated DNR appears to be more promising as a nanodrug compared with free DNR currently used in cancer therapy.

Our results with tumor spheroids show that BPMO is efficiently taken up into the spheroids. In the chicken egg tumor model, we observed dramatic accumulation of BPMO in the tumor after intravenous injection. This was further confirmed by making thin sections of the tumor and analyzing by confocal microscopy. No accumulation of BPMO was seen with liver, spleen or heart. The tumor accumulation was observed three days after the injection. We believe that BPMO‘s prolonged blood circulation contributes to tumor accumulation through the EPR (enhanced permeability and retention) effect.^25^, ^38^

In summary, our results point to attractive features of BPMO as a drug delivery vehicle for cancer therapy. The use of BPMO as nanovector improves efficacy of DNR presumably by improving its biodistribution and pharmacokinetics in the organism. At the same time, adverse effect of free DNR on vital organs such as lung can be minimized by using DNR‐loaded BPMO.

## Conclusion

We have synthesized biodegradable PMO nanoparticles containing tetrasulfide bonds, which can be degraded in a reducing environment. We showed that BPMO can be degraded completely after incubation with glutathione. We used two different models, tumor spheroids and chicken egg tumor model to evaluate effect of BPMO and DNR‐loaded BPMO in biological systems. Of particular note is excellent tumor accumulation of BPMO in the chicken egg tumor model, as confocal microscopy showed specific accumulation in the tumor. Loading DNR into BPMO increased the efficacy of this anticancer drug and decreased toxic side‐effect on other organs raising the possibility that DNR−BPMO has a potential to be used in the clinic.

## Experimental Section

***Characterization***: SEM was performed on a JEOL JSM‐75FCT 200 instrument. TEM was performed on a JEOL JEM‐2100F instrument. Low‐pressure N_2_ adsorption measurements were carried out on the Micromeritics volumetric gas adsorption analyser (3‐FLEX Surface Characterization). A liquid nitrogen bath was used for measurements at 77 K. Helium was used as estimation of dead space. Ultrahigh‐purity‐grade N_2_, and He (99.999 % purity) were used throughout adsorption experiments. Thermal gravimetric analysis (TGA) was performed using a TA Instruments Q‐500 thermal gravimetric analyser under airflow with temperature ramp of 5 °C min^−1^. Fourier transform infrared (FT‐IR) spectra were measured on a Bruker Vertex 70 FT‐IR spectrometer using potassium bromide pellets. DLS was performed using a Zetasizer μ V Malvern apparatus. Raman spectra were recorded on a XploRA PLUS HORIBA Scientific Raman microscope using a He−Ne laser emitting at 532 nm. All confocal laser microscopy images were collected on an Nikon A1R confocal laser microscope.

***Synthesis of biodegradable PMO***: Synthesis of BPMO was slightly modified from a reported procedure.^25^ In particular, RBITC was attached into the framework by co‐synthesis method: 2.5 mg of RBITC was dissolved in ethanol (EtOH, 5 mL) and then 3‐aminotriethoxysilane (APTES, 6 μ L, 2.6×10^−2^ mmol) was added. After stirring this solution for 30 minutes, 1,2‐bis(triethoxysilyl)ethane (300 μ L, 0.8 mmol) was added for further 5 minutes. Simultaneously, a mixture of CTAB (250 mg, 0.7 mmol), distilled water (120 ml) and NaOH (8 M, 219 μ L) was stirred vigorously and heated to 80 °C. Once the temperature of the CTAB solution reach stability, the silane containing solution was added dropwise into the flask. Then bis[3‐(triethoxysilyl) propyl] tetrasulfide (100 μL, 0.2 mmol) was added immediately. To modify the surface of the nanoparticle, 3‐(trihydroxysilyl) propyl methyl phosphonate (2 M, 315 μL) was added to the solution after 15 minutes (zeta potential −31.27 mV). In the case of positively charged BPMO, 120 μL of N‐(2‐aminoethyl)‐3‐aminopropyltrimethoxysilane was added instead of phosphonate compound to get 22.53 mV zeta potential value. After condensation at 80 °C for 2 hours, the PMO material was recovered by centrifugation (30 min at 14 krpm) and washed twice with EtOH. To remove CTAB from the pores, the particles were refluxed overnight in ethanolic solution of ammonium nitrate (0.3 g in 50 mL). The particles were then purified by washing with EtOH (3 times) followed by dessication. The material was stored at room temperature for further characterization and activity evaluation.

***Degradation experiments***: The *in vitro* degradation behavior of BPMO was assessed in both PBS and SBF solution. Specifically, pristine BPMO was dispersed into two kinds of PBS solutions: pure PBS (pH 7.4) and reducing PBS (pH 7.4, GSH 10 mM) at 0.1 mg.mL^−1^. The higher GSH concentration in cytosol (2–10 mM) than in extracellular condition (2–10 μM) generates reducing intracellular microenvironment.^39^ The degradation behavior of BPMO in SBF solution was also evaluated under identical experimental conditions to that above at a concentration of 0.1 mg.mL^−1^. All the solutions were mixed at 37 °C under magnetic stirring. Small fractions of degraded medium were sampled for TEM observations at given period of time. *In vitro* degradation profiles and microstructure changes in BPMO were assessed by TEM.

***Drug loading***: Daunorubicin (DNR) was chosen as anticancer drug in the experiments. 3 mg of BPMO were added into 0.1 M NaHCO_3_ aqueous solution (400 μL) and stirred overnight at 4 °C in cold room. Activated BPMO were centrifuged at 14 krpm for 30 min and then suspended in 355 μL of Milli‐Q water prior to adding 45 μL of DNR solution (10 mg.mL^−1^). The suspension was stirred overnight at 4 °C. DNR‐loaded BPMO were then collected by centrifugation (14 krpm for 30 min), washed with Milli‐Q water and finally stored for further experiments. The supernatant was transferred to Eppendorf tube and filled up to 1 mL with water for fluorescence measurement. The loading efficiency was analysed by fluorescence measurements of the supernatants to determine the mass of encapsulated DNR. A calibration curve of free DNR was prepared under the same conditions in order to determine the drug encapsulation rate. Measurements were taken at 480 nm (excitation) and 560 nm (emission). The calculated drug loading capacity was 12.04 wt%.

***Tumor spheroids culture***: Human ovarian cancer cells OVCAR8 were cultured on 100 mm^2^ culture dish in RPMI1640 medium supplemented with 10 % FBS and 1 % penicillin/streptomycin at 37 °C. For spheroid formation, 1.0×10^4^ of OVCAR8 cells were inoculated on PrimeSurface96 U culture plate (MS‐9096U, Sumitomo Bakelite Co., LTD., Japan) and cultured for 7 days. Tumor spheroids with diameter of around 350 μm were generated.

***Uptake of BPMO by 3D tumor spheroid***: Rhodamine B‐labelled BPMO were added to tumor spheroids and incubated for 24 h. The spheroids were collected into an Eppendorf tube and centrifuged. The supernatant was removed, and spheroids were washed with ice‐cold PBS. They were then centrifuged at 1500 rpm for 5 min and fixed overnight with 4 % paraformaldehyde at 4 °C. Spheroids were then washed with ice‐cold PBS and treated with 99.8 % methanol for 30 min at −80 °C. After washing, spheroids were stained by using Hoechst 33258 solution for 30 min in dark to show nuclei. The BPMO biodistribution was finally observed by using a confocal microscope.

***Evaluation of 3D tumor spheroid growth***: BPMO (10 μg), free DNR (1.2 μg), DNR‐loaded BPMO (10 μg) were incorporated into 96‐well‐plate with ovarian tumor spheroid in 100 μL culture medium at equivalent drug concentration according to fluorescence measurements. For each group, experiment was replicated three times. Plates were incubated at 37 °C for 7 days and the culture medium was changed every two days. To monitor the tumor spheroid growth, optical images were taken and volume (V) calculated using the following formula:

$$V=0.5\times \mathrm{longest}\;\mathrm{diameter}\times \left(\mathrm{shortest}\;\mathrm{diameter}\right){}^{2}$$

***Ovarian cancer tumor formation on CAM***: Fertilized chicken eggs were purchased from Goto farmer in Gifu prefecture and incubated for 10 days at 37.5 °C in a 60 % humidity with rotation. The thick blood vessels of chicken eggs were checked under the light and a window was cut using grinder. OVCAR8 (Human ovarian cancer cells) cells were grown on the 100 mm^2^ culture plates in RPMI1640 supplemented with 10 % heat‐inactivated FBS and 1 % penicillin/streptomycin for transplantation. To transplant OVCAR8 cells, a sterile Teflon ring was set on a part of the blood vessel and 2.0×10^6^ of OVCAR8 cells were transplanted on the CAM inside Teflon ring. OVCAR8 tumor rapidly formed 3 days after transplantation and was allowed to develop for a total of 13 days. All chicken egg experiments were approved by the Kyoto University Animal Research Committee and were performed in compliance with the committee guideline. *In ovo* experiments do not require any special additional allowance as long as the embryos are sacrificed before hatching as is done in this study.

***Investigation of BPMO nanoparticles biodistribution***: On the 13th day chicken eggs were selected with OVCAR8 tumor on CAM. BPMO (0.2 mg.100 μL^−1^) were intravenously injected. 2 days after injection, tumor and organs were cut out and observed using a fluorescent stereomicroscope. In addition, thin sections of tumor and organs were prepared to evaluate precisely the biodistribution as follows. Tumor and organs were fixed overnight with 4 % paraformaldehyde at 4 °C. After washing with ice‐cold PBS, the tissue was treated with methanol for 30 min at −80 °C. After washes with ice‐cold PBS, tumor and organs were treated overnight with 20 % sucrose solution at 4 °C. Tumor and organs were then frozen prior to being sliced with 30 μm in thickness by the cryomicrotome. These thin sections were then stained with Hoechst 33258 solution (1 mg.mL^−1^), which was 5‐fold diluted into PBS, for 30 min in dark. The BPMO biodistribution on thin sections was observed by using a confocal laser microscope.

***Evaluation of tumor elimination effect of DNR‐loaded BPMO***: Free DNR or DNR‐loaded BPMO were intravenously injected into chicken eggs with OVCAR8 tumor on CAM (day 13). Tumor elimination effect was observed by fluorescent stereomicroscope owing to GFP and rhodamine B intrinsic fluorescence. Tumor and organs were cut out and tumor weight was measured for each DNR drug or DNR‐loaded BPMO treatment. The data are presented as mean ± standard error of the mean (SEM) of three replicates. DNR or DNR‐loaded BPMO biodistribution were observed by fluorescent stereomicroscope.

## Supporting information

As a service to our authors and readers, this journal provides supporting information supplied by the authors. Such materials are peer reviewed and may be re‐organized for online delivery, but are not copy‐edited or typeset. Technical support issues arising from supporting information (other than missing files) should be addressed to the authors.

Supplementary
