# Supplementary material for: Biodegradable Periodic Mesoporous Organosilica (BPMO) Loaded with Daunorubicin: A Promising Nanoparticle‐Based Anticancer Drug
Source: ChemMedChem. 2020 Feb 11;15(7):593–9. doi: 10.1002/cmdc.201900595 (PMC7187469; doi:10.1002/cmdc.201900595)
Supplement: Supplementary file 1 — Supplementary [file CMDC-15-593-s001.pdf]

CHEMMEDCHEM

Supporting Information

Biodegradable Periodic Mesoporous Organosilica (BPMO) Loaded with Daunorubicin: A Promising Nanoparticle-Based Anticancer Drug

Ngoc Xuan Dat Mai, Albane Birault, Kotaro Matsumoto, Hanh Kieu Thi Ta, Soontaree Grace Intasa-ard, Kendall Morrison, Phan Bach Thang, Tan Le Hoang Doan, and Fuyuhiko Tamanoi*© 2020 The Authors. Published by Wiley-VCH Verlag GmbH & Co. KGaA. This is an open access article under the terms of the Creative Commons Attribution License, which permits use, distribution and reproduction in any medium, provided the original work is properly cited.

Author Contributions

F.T. Conceptualization:Lead; Data curation:Lead; Formal analysis:Lead; Funding acquisition:Lead; Investigation:-Lead; Methodology:Lead; Project administration:Lead; Resources:Lead; Software:Lead; Supervision:Lead; Validation:Lead; Visualization:Lead; Writing - Original Draft:Lead; Writing - Review & Editing:Lead
N.M. Conceptualization:Equal; Data curation:Equal; Formal analysis:Equal; Investigation:Equal; Writing - Original Draft:Equal; Writing - Review & Editing:Equal
A.B. Conceptualization:Equal; Data curation:Equal; Formal analysis:Equal; Investigation:Equal; Writing - Original Draft:Equal; Writing - Review & Editing:Equal
K.M. Conceptualization:Equal; Data curation:Equal; Formal analysis:Equal; Investigation:Equal; Supervision:Equal; Writing - Original Draft:Equal; Writing - Review & Editing:Equal
H.T. Data curation:Equal; Formal analysis:Equal; Investigation:Equal; Writing - Review & Editing:Equal
S.I.-a. Data curation:Equal; Formal analysis:Equal; Investigation:Equal; Writing - Review & Editing:Equal
K.M. Formal analysis:Equal; Writing - Review & Editing:Equal
P.T. Data curation:Equal; Formal analysis:Equal; Investigation:Equal; Writing - Review & Editing:Equal
T.D. Conceptualization:Equal; Data curation:Equal; Formal analysis:Equal; Funding acquisition:Equal; Investigation:Equal; Writing - Review & Editing:Equal

Supporting Information

Table S1. IR band assignments of BPMO shown in Figure 1-e ([1-3])

**Band position [cm^{-1}]**	**Band assignment**
3436	C-H stretching
2925	C-H
1269	CH_2 wagging in -CH_2-S-
1159	Si-O-Si
1103	Si-O-CH_2CH_3
1035	Si-O-Si
909	C-H
778	Si-C stretching
696	C-S
456	Si-(O-CH_2CH_3)$_3$ symmetric deformation

Table S2. Average of tumor weight 3 days after injection

**Sample**	**Control**	**Free DNR**	**DNR-BPMO**
**Weight (mg)**	55.67 ± 1.19	28.46 ± 2.70	3.03 ± 1.01

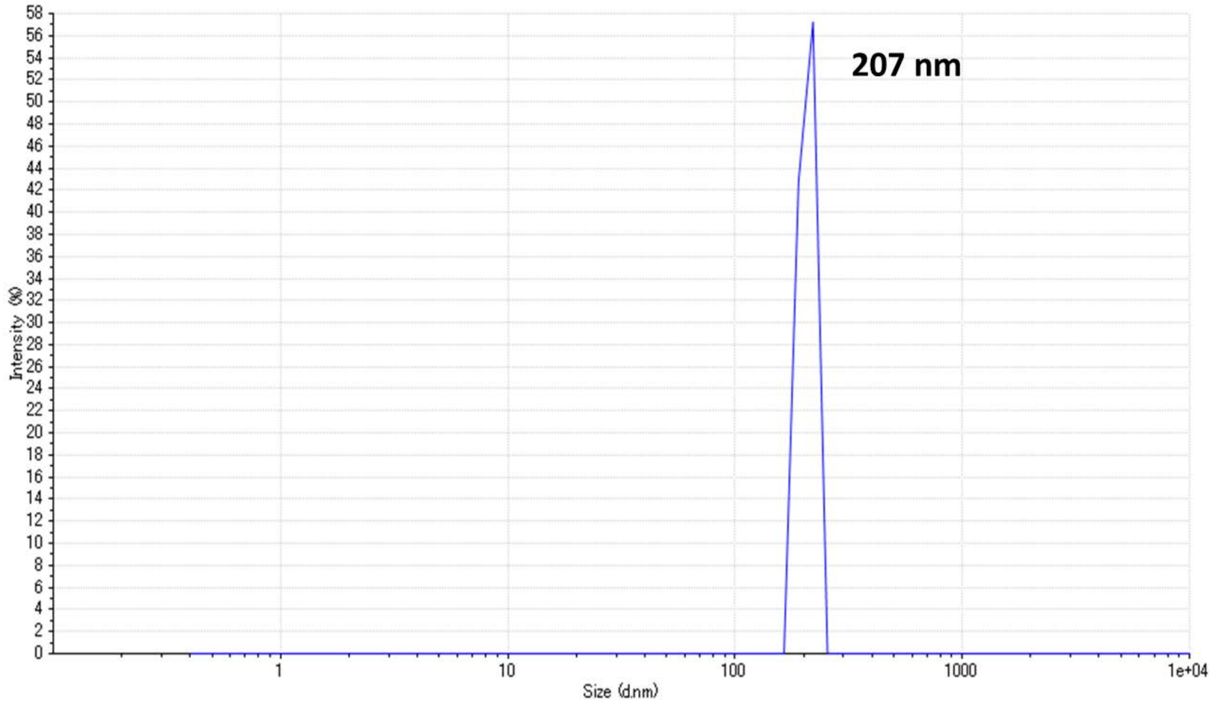

Figure S1. DLS of BPMO was analyzed by Zetasizer μV Malvern apparatus.

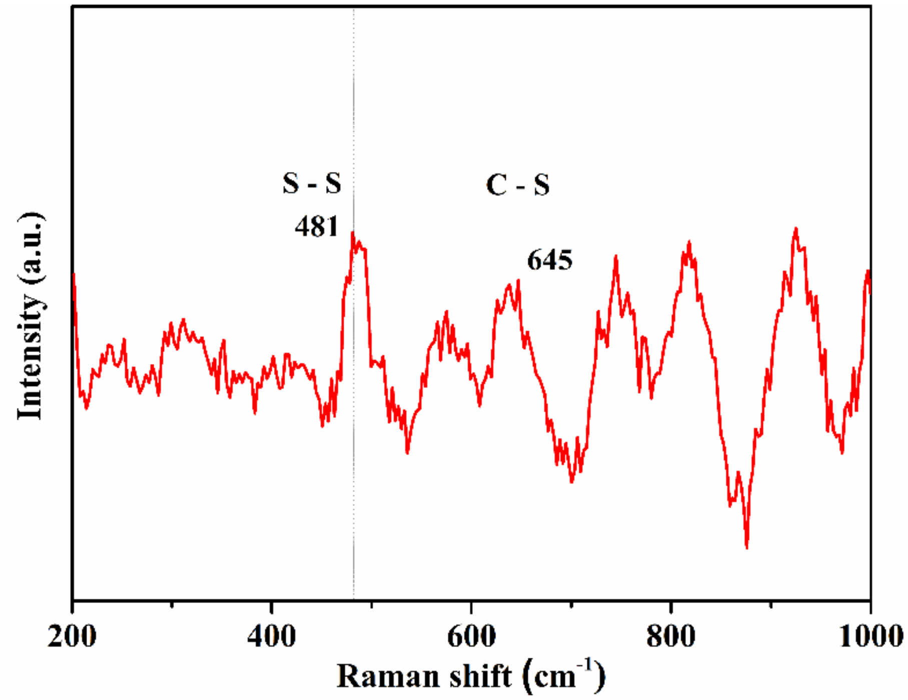

Figure S2. Raman spectrum of BPMO was analysed by XploRA PLUS HORIBA Scientific Raman microscope.

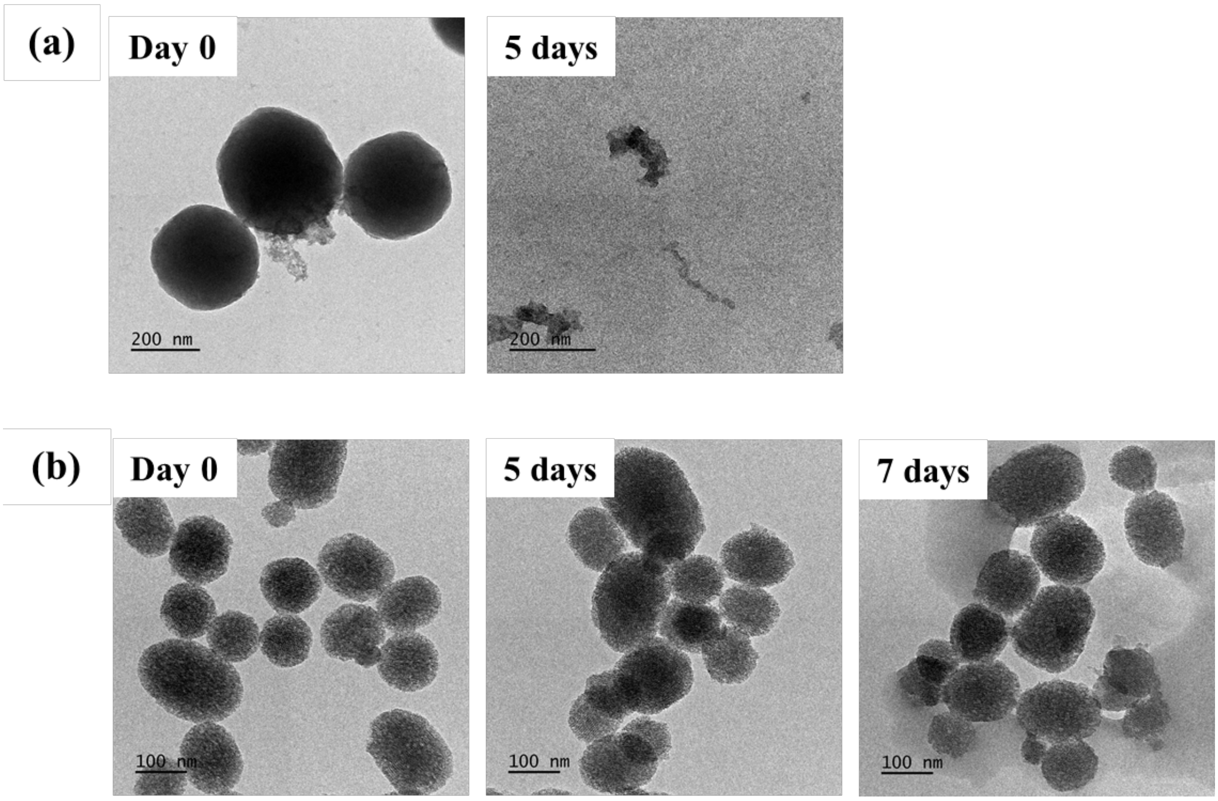

Figure S3. Degradation of BPMO and MSN by GSH. TEM of (a) BPMO and (b) MSN nanoparticles after dispersion in PBS with GSH (10 mM) for various times.

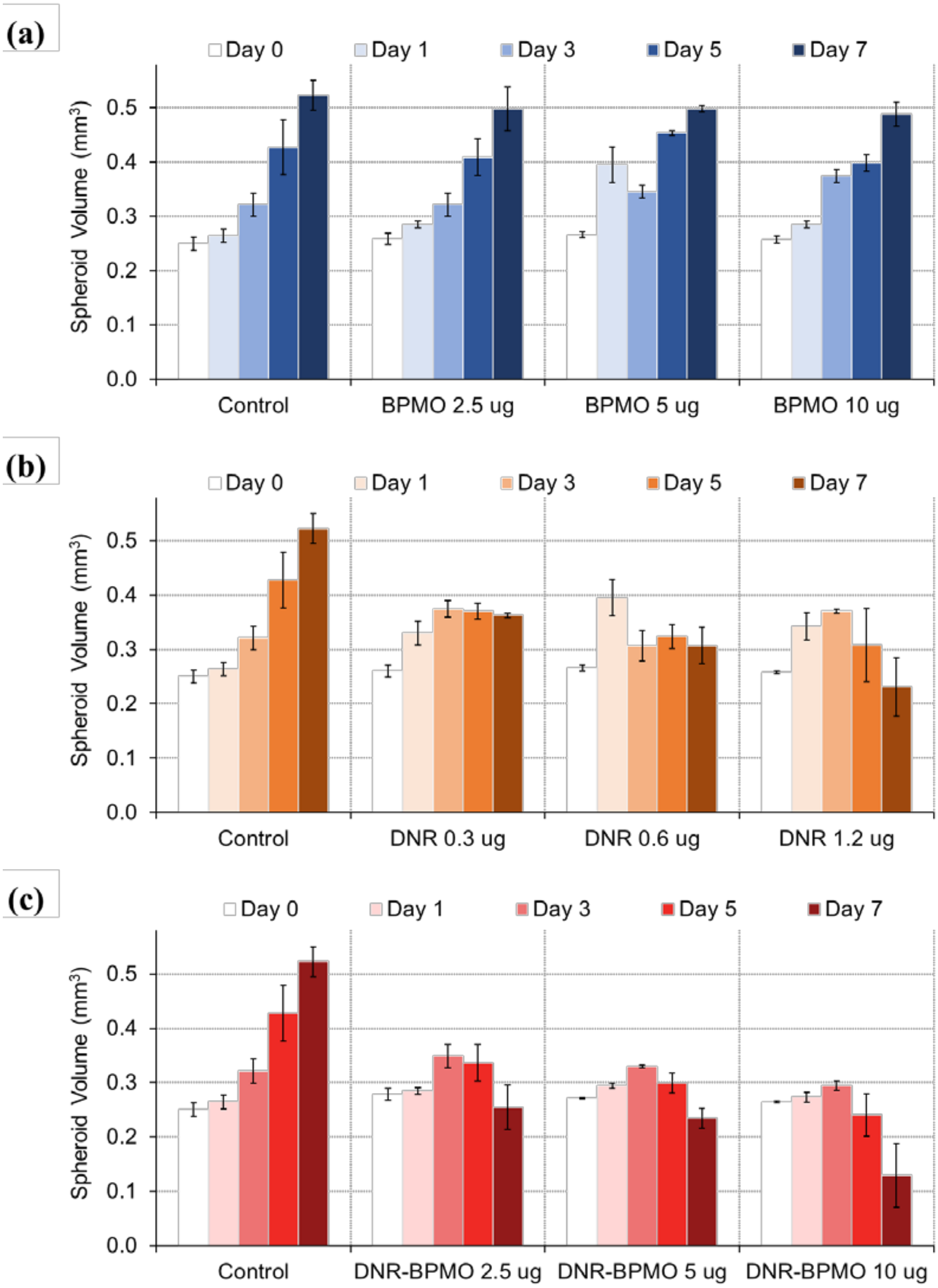

Figure S4. Spheroid volume calculated after treatment with no injection (Control), (a) BPMO (2.5, 5 and 10 µg), (b) free DNR (0.3, 0.6 and 1.2 µg) or (c) DNR-BPMO (2.5, 5 and 10 µg) for 7 days. Error bars show standard error.

References

[1] N. Lu, Y. Tian, W. Tian, P. Huang, Y. Liu, Y. Tang, C. Wang, S. Wang, Y. Su, Y. Zhang, J. Pan, Z. Teng, G. Lu, *ACS Appl. Mater. Interfaces.* **2016**, *8*, 2985.

[2] D. Zhu, W. J. van Ooij, *J. Adhes. Sci. Technol.* **2002**, *16*, 1235.

[3] J. H. Kim, B. Fang, M. Y. Song, J.-S. Yu, *Chem. Mat.* **2012**, *24*, 2256.
